# Establishing the HLS-M-Q18 short version of the European health literacy survey questionnaire for the Malaysian context

**DOI:** 10.1186/s12889-020-08704-7

**Published:** 2020-04-28

**Authors:** Emma Mirza Wati Mohamad, Manimaran Krishnan Kaundan, Mohammad Rezal Hamzah, Arina Anis Azlan, Suffian Hadi Ayub, Jen Sern Tham, Abdul Latiff Ahmad

**Affiliations:** 1 Universiti Kebangsaan Malaysia, 43600 UKM Bangi, Selangor Malaysia; 2 Ministry of Health Malaysia, 40170 Setia Alam, Selangor Malaysia; 3 Universiti Malaysia Perlis, 01000 Kangar, Perlis Malaysia; 4 Sunway University Malaysia, 47500 Petaling Jaya, Selangor Malaysia; 5 Universiti Putra Malaysia, 43400 Serdang, Selangor Malaysia

**Keywords:** Health literacy, HLS-M-Q18, Short version instrument, HLS-EU-Q47, Instrument item reduction

## Abstract

**Background:**

The European Health Literacy Survey Questionnaire (HLS-EU-Q47) is becoming a widely used tool to measure health literacy (HL), including in Malaysia. There are efforts to reduce the 47-item scale to parsimonious short item scales that still reflect the assumptions and requirements of the conceptual model. This study used confirmatory factor analysis to reduce the 47-item scale to a short scale that can offer a feasible HL screening tool with sufficient psychometric properties.

**Methods:**

A cross-sectional survey was conducted on the Malaysian population based on ethnic distribution to ensure that the short version instrument reflects the country’s varied ethnicities. The survey was administered by well-trained interviewers working for the Ministry of Health Malaysia. A total of 866 responses were obtained. Data was analysed using multi-factorial confirmatory factor analysis (CFA) with categorical variables.

**Results:**

The analysis resulted in a satisfactory 18-item model. There were high correlations among the 18 items. The internal consistency reliability was robust, with no floor/ceiling effects. These results represented equivalence and consistency among the responses to items, suggesting that these items were homogenous in measuring Malaysian health literacy. The strong convergent and discriminant validity of the model makes the proposed 18 items a suitable short version of the health literacy instrument for Malaysia.

**Conclusions:**

The researchers propose the 18-item instrument to be named HLS-M-Q18. This short version instrument may be used in measuring health literacy in Malaysia as it achieved robust reliability, structural validity and construct validity that fulfilled goodness-of-fit criteria.

## Background

Health literacy does not only measure the ability to seek information on the importance of health. It is also an asset to the individual as it empowers their health status continuously. Health literacy has the potential to be a risk factor to individuals who suffer from illness or disease if their level of literacy is low. This could further deteriorate their health status [1]. Health literacy is a necessity in ensuring better health levels. In general, the information seeking activity on health will lead to an increase in an individual’s health literacy level [2, 3].

In the Malaysian context, an extensive health literacy study has not been conducted. Even so, there are more segmentised studies that consist of specific groups with various forms of measurements [4, 5].

It is thus vital for local authorities such as the Health Ministry of Malaysia to explore and monitor the Malaysian health literacy level at a greater and inclusive scale. It is only from this data that the ministry can initiate projects and activities to increase the health literacy level of Malaysians cutting across age, education level and socioeconomic status.

To achieve this, it was suggested that the measurement of health literacy be incorporated into the Malaysian National Health Morbidity Survey (NHMS) 2019 to measure health literacy levels among Malaysians. The HLS-EU instrument was developed by Sorenson et al. based on a systematic review on defining and conceptualising the health literacy framework [6]. The Sorensen systematic review evaluated 17 definitions and 12 models of health literacy concepts. They have proposed an integrative conceptual model that consists of 12 dimensions – knowledge, motivation and ability to access, understand, appraise and apply health information in the context of health care, disease prevention and health promotion.

However, the HLS-EU contains 47 items (HLS-EU-Q47) and is rather extensive to be adapted into the NHMS 2019. Thus, the objective of this study was to shorten the instrument into a concise set of items for use in the survey.

## Methods

### Study design

An earlier population-based cross-sectional study [7] has validated the suitability of HLS-EU-Q47 for the Malaysian context. The present study also employed a cross-sectional design on the Malaysian population based on ethnic distribution to ensure that the short version instrument reflects the country’s varied ethnicities. Participants were between the age of 18 to 60 years old. Adapted measures from the HLS-EU-47 [8] validated in English and Malay in a previous study [7] were utilised. However, a 3-level face validation process was conducted and our researchers have restructured the sequence of some items to allow for better comprehension and reduce confusion for respondents. Some items were also reworded upon recommendation by health education experts through the face validation stage.

The survey was administered by well-trained interviewers working for the Ministry of Health Malaysia. Three states were selected (Selangor, Kuala Lumpur and Sarawak) to represent the distribution of multiple ethnicities, as well as the distribution of urban and rural areas. The selection of areas were made based on referral and advice by the District of Jurisdiction Malaysia, Rural Master Plan Malaysia, and previous literature [9].

### Ethical approval

This study was submitted for ethical review and received ethical approval from the National Medical Ethics Committee Malaysia which governs all medical/health related research in Malaysia. The National Medical Research Registration (NMRR) ID obtained for this study is 41882 and approval number is NMRR-18-1320/41882. The NMRR approval is the only ethical approval needed as this project was submitted under the Ministry of Health Malaysia and the National Medical Ethics Committee Malaysia is the Ministry’s Institutional Regulatory Board for Ethical Approval.

All respondents were above 18 years old and therefore involved no minors. All respondents also signed a written consent form clearly stating their rights and nature of participation in the study before being asked to answer the survey. This consent form was also submitted and approved by the National Medical Ethics Committee Malaysia.

### Sampling method

**Fig. 1:**
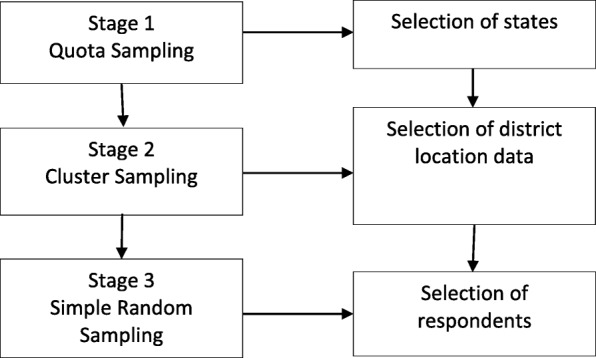
The multi-stage random sampling procedure

Multi-stage random sampling was used in this study. Specifically, there were three stages involved, utilising several sampling techniques (quota sampling, cluster sampling and simple random sampling) to allow random data collection. The three stages are as in Fig. 1. The researchers made the decision to prioritise an inclusive Malaysian sample based on ethnicity and urban/rural strata due to constraints in resources. This was to ensure that the smaller groups were adequately represented in the sample. The list of states, ethnicities and urban/rural distribution required for this study are as presented in Table 1.

**Table 1:** Sample Distribution

State	Locality	Ethnicity	n	Area
Selangor	Urban	Malay	299	Shah Alam Seksyen 1–5 Seksyen 31 Seksyen U9 Seksyen U13
(6.298mil = 58.2%)	(93.3%)	Chinese	103	
*n* = 466	*n* = 435	Indian	33	
	Rural	Malay	21	Hulu Langat Sesapan Kelubi Village Sesapan Batu Rembau Village Sungai Jai Village Taman Indah PKNS
	(6.7%)	Chinese	7	
	*n* = 31	Indian	3	
Kuala Lumpur	Urban	Malay	82	Segambut, Lembah Pantai
(1.782mil = 16.5%)	(100%)	Chinese	38	
*n* = 132	*n* = 132	Indian	12	
Sarawak	Urban	Bumiputera	91	Kuching
(2.741mil = 25.3%)	(57.8%)	Chinese	26	
*n* = 202	*n* = 117	Indian	0	
	Rural (42.2%)	Bumiputera	66	Sarikei (Maradong), Samarahan (Simunjan)
	*n* = 85	Chinese	19	
		Indian	0	

Location of study is determined by population density and racial distribution

In stage 1, quota sampling based on ethnicities and urban/rural distribution were used to select three Malaysian states. Ethnic distribution should be a standard in sampling multiracial populations to ensure inclusivity of the sample [10]. States from both Peninsular Malaysia and Borneo were selected to represent the diverse ethnicities in Malaysia. For the purpose of urban and rural distribution, Kuala Lumpur was selected to represent the urban area majority. This is justified as Kuala Lumpur has the highest urban population in Malaysia. Sarawak was selected to represent the rural distribution, as well as to give more balanced representation of the minority ethnic groups. Selangor represents both urban and rural areas and has a balanced ratio in ethnic group distribution. Selection of the three states was decided upon discussion between researchers and the Ministry of Health Malaysia.

In stage two, researchers utilised cluster sampling to determine districts of choice. District sampling for Selangor was determined based on the demographic distribution list published by the Selangor Economic Development Unit, as well as extant literature [9]. For selection of districts in Kuala Lumpur, researchers used data provided by the Department of Information, Ministry of Communications and Multimedia Malaysia; and for selection of districts in Sarawak, the selection of districts was guided by data provided by the State Director of the Fire and Rescue Department. The definitions of rural and urban were determined by the National Department of Statistics and The Rural Master Plan, published by the Ministry of Rural Development Malaysia.

In stage three, respondents were selected based on a simple random sampling technique based on several criteria (i.e., Malaysian, aged 18 and above, resident in the chosen state, able to make health decisions for themselves). This is also the same protocol criteria used by the Asian Health Literacy Consortium. Only one person was selected in each household, in which the eldest household member would be chosen if there was more than one person who met the respondent selection criteria (similar technique used in literature) [11].

Sample size was calculated based on the minimum requirement for CFA rule of ten [12]. Based on the 47 items, the study would need a minimum of *N* = 470 in order to perform CFA with a 95% confidence level. However, the researchers decided to increase the number of respondents to improve the confidence interval, thereby reducing the margin of error [13].

Data collection was conducted between 25th June 2018 to 14th July 2018, involving 18 enumerators. Respondents took an average of 30–40 min to fill in the questionnaire.

### Questionnaire and measurement

The health literacy survey questionnaire (HLS-EU-Q47) contained 47 items measuring health literacy. The perceived difficulty of each item was rated on a 4-point Likert scale which ranged from 1 = very difficult to 4 = very easy. The HLS-EU-Q47 was developed based on a conceptual model of health literacy which measures four individual competencies (the ability to access, understand, appraise, and apply health information) across three domains i.e.: health care, disease prevention and health promotion [8].

### Participant and data collection procedure

The enumerators went from house to house within the selected areas and provided the self-reported questionnaire to be answered. A consent form was filled in and obtained from each participant. Although the researchers aimed to collect responses from 800 respondents, a total of 866 complete responses with no missing data were obtained throughout the data collection period and analysed. All trained interviewers wore the Ministry of Health uniform and identity card to avoid misunderstanding and protect the interest of both researchers and respondents.

### Data analysis

#### Selection of items and validity analyses

The analysis procedures utilised in this study closely follow those conducted by previous studies [14–16] to allow for flexibility in exploring the relationships between variables in the model, not limited to model and item evaluation. To establish construct validity, CFA was conducted separately for the three health literacy domains of health care, disease prevention, and health promotion. The fit of the data to the model was examined using goodness-of-fit indices, including (i) absolute model fit: root mean square error of approximation (RMSEA) and goodness-of-fit index (GFI); (ii) incremental fit: adjusted goodness-of-fit index (AGFI), comparative fit index (CFI), incremental fit index (IFI), and normal fit index (NFI).

#### Reliability analyses

Internal consistency was tested with Cronbach’s alpha, and values greater than or equal to 0.7 indicate satisfactory reliability.

## Results

Table 2 shows that out of the total 866 respondents, 303 were males (35.0%) and the remaining 563 were females (65.0%). As for the age of the respondents, the average year of birth was 1984 and this subsequently indicates that the majority of the respondents consisted of the Generation Y (1977–1994) group, representing half of the sample at 442 respondents (51.0%).

**Table 2:** Characteristics of participants

Variables	n	%
Gender		
Male	303	35
Female	563	65
Year of birth	Min = 1984.37 (12.07)	
Baby Boomers (1950–1965)	101	11.7
Generation X (1966–1976)	111	12.8
Generation Y (1977–1994)	442	51.0
Generation Z (1995–2012)	212	24.5

### Selection of items and validity of health literacy model

A CFA was conducted to produce a model with good fit indices and a set of items which are suitable for the measurement of health literacy. To validate the construct, the researchers referred to the CR, AVE, MSV, and ASV values [17]. CR values above 0.7 and AVE values exceeding 0.5 indicate good convergent validity whereas MSV and ASV values that are smaller than AVE indicate good discriminant validity of the constructs.

The test results indicate that the model fit did not adhere to goodness-of-fit indices (Table 3). The analysis indicated that the first tier of CFA resulted in χ2 = 21,718.433, df = 6867, *p* = 0.000, χ2/df = 8.007, GFI = 0.664, CFI = 0.683, NFI = 0.654, TLI = 663. and RMSEA = 0.090. Meanwhile the CMIN/df showed that the value exceeded 5. These results illustrated that the model was not compatible with the data of the study. Nevertheless, in the aspect of construct validity, the model had produced good convergent and discriminant validity (Table 4).

**Table 3:** Goodness of fit indices for the 47-item model and the modification model

Goodness of fit indices	Overall 47 Items (HLS-EU)	Modification model (final model)
CMIN	8159.118	395.956
CMIN/DF	8.007	3.272
Df	1019	121
p	0.000	0.000
GFI	0.664	0.950
NFI	0.654	0.937
IFI	0.684	0.955
CFI	0.683	0.955
TLI	0.663	0.943
RMSEA	0.090	0.051

**Table 4:** Overall model for convergent and discriminant validity

Health literacy domains	CR	AVE	MSV	ASV	HC	DP	HP
HC	0.96	0.866	0.73	0.708	0.931		
DP	0.981	0.928	0.748	0.716	0.827	0.96	
HP	0.971	0.895	0.748	0.740	0.856	0.865	0.946

*HC* Healthcare, *DP* Disease Prevention, *HP* Health Promotion, *CR* Composite Reliability, *AVE* Average Variance Extracted, *MSV* Maximum Shared Variance, *ASV* Average Shared Variance

To improve the model fit, a modification model was developed to determine the measurable items that complied with goodness of fit indices and displayed good convergent and discriminant validity.

To determine whether the model developed met the goodness-of-fit indices, modifications were made to the original model. The result in Table 3 exhibited that the modification model fulfilled the goodness-of-fit indices with χ2 = 395.956, df = 121, *p* = 0.000, χ2/df = 3.272, GFI = 0.950, CFI = 0.955, NFI = 0.937, TLI = 0.943 and RMSEA = 0.051. This finding further illustrated that the items used to measure the health literacy model were suitable with 18 items with the data of the study.

Furthermore, the modification model showed strong construct validity in the aspects of convergent and discriminant validity (Table 5). Even so, the developed model would not be able to retain the original sub-domains in the original 47-item health literacy model from previous survey tools [6].

**Table 5:** Convergent and discriminant validity for the modification model

Health literacy domain	CR	AVE	MSV	ASV	DP	HC	HP
DP	0.982	0.948	0.566	0.512	0.974		
HC	0.987	0.962	0.801	0.630	0.677	0.981	
HP	0.940	0.842	0.801	0.68	0.752	0.895	0.917

*DP* Disease Prevention, *HC* Healthcare, *HP* Health Promotion, *CR* Composite Reliability, *AVE* Average Variance Extracted, *MSV* Maximum Shared Variance, *ASV* Average Shared Variance

### Instrument reliability

Table 6 illustrates the summary for instrument validity which refer to the Cronbach’s alpha values. All of the health literacy domains in the 18-item model indicate reliability levels (more than 0.7) across the three domains (healthcare, health promotion, and disease prevention).

**Table 6:** Cronbach’s alpha values

Domain	Positioning of HLS-47 in the questionnaire	HLS-EU (47)	Item-total correlation	Alpha Cronbach if item deleted	Alpha Cronbach values
Healthcare	Q1.36	15	.42	.797	0.798
	Q1.30	11	.601	.754	
	Q1.35	7	.529	.772	
	Q1.28	16	.587	.759	
	Q1.27	9	.598	.756	
	Q1.31	6	.589	.758	
Health promotion	Q1.21	39	.511	.778	0.798
	Q1.39	41	.569	.764	
	Q1.40	42	.63	.749	
	Q1.41	47	.497	.781	
	Q1.44	37	.520	.775	
	Q1.47	44	.598	.757	
Disease prevention	Q1.4	21	.414	.844	0.835
	Q1.8	18	.510	.830	
	Q1.12	2	.734	.782	
	Q1.11	19	.661	.797	
	Q1.1	27	.729	.784	
	Q1.14	25	.624	.805	
HLS-M-Q18					0.906

The construct validity has also been reviewed by observing the Pearson correlation values of each item against the total scores of the measured variables. Based on previous literature, correlation values above 0.25 are good and signal high construct validity [17]. Correlation scores can be categorised into 3 different stages; low (0.10 to 0.29), average (0.30 to 0.49) and high (0.50 to 1.00) [18]. The result of this study has shown that the correlation value of each item with their overall domain value is good (0.414 to 0.734).

## Discussion

The purpose of this study was to produce a set of items suitable to measure health literacy in Malaysia through the adaptation of the HLS-EU model. Structural equation modelling was used to conduct a confirmatory factor analysis. The results showed that the HLS-EU-Q47 was not an ideal fit to the Malaysian data. However, a modified model containing 18 items was found to be valid and reliable in measuring health literacy in Malaysia. The 18-item model, the HLS-M-Q18, showed satisfactory fit indices as well as good convergent and discriminant validity.

### Item selection and structural validity

The HLS-M-Q18 was validated among 866 respondents. CFA is an important step to confirm the domains of health literacy and define its structure. The HLS-EU-Q47 instrument was designed to measure the multiple aspects of health literacy, namely accessing, understanding, evaluating and applying health information in the contexts of health care, disease prevention and health promotion. The initial model (HLS-EU-Q47) showed poor goodness-of-fit and was therefore modified to achieve acceptable fit indices.

In evaluating model fit, guidelines suggested by previous literature were adhered [14]. The researchers constructed three modification models and the results were satisfactory with an 18-item model. There were high correlations among the 18 items, ranging from 0.414 to 0.734 using Pearson Correlation.

The modification model, HLS-M-Q18, consisted of 18 items that retained 9 out of the 12 original domains of the HLS-EU-Q47. For health care, the model retained only three out of the four sub-domains: understanding, appraising and application of information. Three domains were retained for disease prevention and health promotion: access, understanding and appraising of information. The exclusion of three original domains of the HLS-EU-Q47 may have decreased the conceptual representation of the original model but has resulted in a valid and reliable measure with increased utility for the Malaysian population.

The HLS-M-Q18 showed satisfactory convergent and discriminant validity (CR > 0.7, AVE > 0.5, MSV < AVE and ASV < AVE), demonstrating that the items within the sub-domains were adequately related and that the sub-domains were sufficiently different from one another.

### Reliability

The instrument (HLS-M-Q18) was reliable, with high internal consistencies. All sub-scales achieved Cronbach’s alpha of 0.80 and above, and the overall instrument achieved 0.91. These results represented equivalence and consistency among the responses to items of HLS-M-Q18, suggesting that these items were suitable in measuring people’s health literacy in the Malaysian context. The internal consistency reliability was robust, with no floor/ceiling effects. However, the results are different as compared to the previous version of HLS-EU-Q16 [2, 17]. This indicates that the model hinges on the context of the country. The HLS-EU-Q16 did not achieve a good model fit in the Malaysian population. Therefore, the researchers used the HLS-EU-Q47 as a benchmark to shorten its version for the context in Malaysia. This is in line with many other studies that utilised the HLS-EU-Q47 as a benchmark for reducing the items for their respective countries [2, 7, 19].

### Limitations

Sampling for the survey was conducted using a quota sampling procedure that was based on population parameters such as ethnicity and place/ area of residence. Data was collected from three selected states in Malaysia. Due to the random sampling procedure utilised prioritising ethnic group and urban/ rural stratas, this study did not precisely reflect the current gender and age distribution in Malaysia. In this study, 65% of our respondents were women while the Malaysian population estimates women at 49% [20]. The age distribution in this study observed a high proportion of respondents between the ages of 25 to 42 years (51% of the sample) as compared to the Malaysian population which indicates that only 32.9% Malaysians are between the ages of 25 and 44 [20]. The use of the HLS-M-Q18 within larger Malaysian settings would provide further evidence on the validity and reliability of the instrument, particularly with the country’s ethnic, age, gender and social economic composition taken into consideration.

A second limitation of the study is that, as with most short-version instruments, the HLS-M-Q18 may not be able to measure each health literacy domain independently. Rather, it is most appropriate for the measurement of health literacy as a single, unified domain. As a consequence, the shortened instrument may not be suitable for all situations. The validity and reliability of instruments are commonly compromised in their shorter versions. It was therefore important that convergent and discriminant validity of the HLS-M-Q18 were met, as demonstrated in this study.

### Implications

Previous research recommended that the HLS-EU-Q47 be used for a comprehensive measurement of health literacy [7]. Even so, the authors also suggest that cultural settings be considered when adapting the instrument for use, emphasising the importance of testing and validating in a population to ensure suitability. At the same time, a shorter version of the health literacy instrument is preferable due to its practicality of use, especially when used in combination with the measurement of other health variables (such as in the National Health Morbidity survey). With the development of the HLS-M-Q18, health literacy in Malaysia is able to be measured with minimal difficulty and a shorter response time.

## Conclusions

The HLS-M-Q18 model developed through this study has resulted in 18 items measuring health literacy in the Malaysian context. This short version instrument may be used in measuring health literacy in Malaysia as it achieved robust reliability and structural validity that fulfilled goodness-of-fit criteria. However, to improve generalisability of findings, more validation studies should be conducted taking into consideration the limitations of this study.

## Data Availability

The authors do not have permission to release the data. However, the data are available from The Institute for Health Behavioural Research, Ministry of Health, Malaysia upon application.

## Abbreviations

DP: Disease Prevention
HC: Healthcare
HP: Health Promotion
CR: Composite Reliability
AVE: Average Variance Extracted
MSV: Maximum Shared Variance
ASV: Average Shared Variance

## References

[1] Lam MK, Lam LT. Health information-seeking behaviour on the Internet and health literacy among older Australians. EJHI. 2012;7(2):1–7.

[2] Atay E, Göktaş S, Emiral ÖG (2018). The health literacy level and eating behaviours of the teachers working at the city center of Eskisehir Turkey. J Res Med Sci.

[3] Morris NS, Field TS, Wagner JL, Cultrona SL, Roblin DW, Gaglio B, Williams AE, Han PJ, Costanza ME, Mazor KM (2019). The association between health literacy and cancer-related attitudes, behaviors, and knowledge. J Health Commun.

[4] Hamzah SR, Suandi T, Ishak NH (2016). Association between health literacy and demographic factors among adolescents in Malaysia. Paper presented at.

[5] Chan HK, Hassali MA, Lim CJ, Saleem F (2015). Exploring health literacy and difficulty in comprehending paediatric medication labels among caregivers in Malaysia: a pilot study. J Pharm Health Serv Res.

[6] Sorensen K, Van den Broucke S, Fullam J, Doyle G, Pelikan J, Slonska Z, Brand H (2012). Health literacy and public health: a systematic review and integration of definitions and models. BMC Public Health.

[7] Duong TV, Aringazina A, Baisunova G, Nurjannah PTV, Pham KM, Truong TQ, Nguyen KT, Oo WM, Mohamad E, Su TT, Huang HL, Sorensen K, Pelikan JM, Van den Broucke S, Chang PW (2017). Measuring health literacy in Asia: validation of the HLS-EU-Q47 survey tool in six Asian countries. J Epidemiol.

[8] Sorensen K, Van den Broucke S, Pelikan JM, Fullam J, Doyle G, Slonska Z, Kondilis B, Stoffels V, Osborne RH, Brand H (2013). Measuring health literacy in populations: illuminating the design and development process of the European health literacy survey questionnaire (HLS-EU-Q). BMC Public Health.

[9] Froze S, Arif MT, Saimon R (2018). Does health literacy predict preventive lifestyle on metabolic syndrome? A population-based study in Sarawak Malaysia. Open J Prev Med.

[10] Charmaraman L, Woo M, Quach A, Erkut S (2014). How have researchers studied multiracial populations? A content and methodological review of 20 years of research. Cult Divers Ethn Minor Psychol.

[11] Berens EM, Vogt D, Ganahl K, Weishaar H, Pelikan J, Schaeffer D (2018). Health literacy and health service use in Germany. Health Lit Res Pract.

[12] Koran J (2017). Preliminary proactive sample size determination for confirmatory factor analysis models. Meas Eval Couns Dev.

[13] Faber J, Fonseca LM (2014). How sample size influences research outcomes. Dental Press J Orthod.

[14] Duong TVD, Chang PWS, Yang S-H, Chen MC, Chao W-T, Chen T, Chiao P, Huang H-L (2017). A new comprehensive short-form health literacy survey tool for patients in general. Asian Nurs Res (Korean Soc Nurs Sci).

[15] Campbell HS, Hall A, Sanson-Fisher R, Barker D, Turner D, Taylor-Brown J (2014). Development and validation of the short-form survivor unmet needs survey (SF-SUNS). Support Care Cancer.

[16] Jones K, Brennan D, Parker E, Jamieson L (2014). Development of a short-form health literacy dental scale (HeLD-14). Community Dent Oral Epidemiol.

[17] Hair J, Black W, Babin B, Anderson R (2010). Multivariate data analysis.

[18] Nunnally J, Bernstein I (1994). Psychometric theory.

[19] Wångdahl J, Lytsy P, Mårtensson L, Westerling R (2014). Health literacy among refugees in Sweden–a cross-sectional study. BMC Public Health.

[20] Malaysian Department of Statistics. Current population estimates: key statistics. Putrajaya: DOSM; 2019.

